# Selective Pressure and Evolution of SARS-CoV-2 Lineages BF.7 and BQ.1.1 Circulating in Italy from July to December 2022

**DOI:** 10.3390/microorganisms12050908

**Published:** 2024-04-30

**Authors:** Alessandra Lo Presti, Luigina Ambrosio, Angela Di Martino, Arnold Knijn, Luca De Sabato, Gabriele Vaccari, Ilaria Di Bartolo, Stefano Morabito, Anna Teresa Palamara, Paola Stefanelli, on behalf of the Italian Genomic Laboratory Network

**Affiliations:** 1 Department of Infectious Diseases, Istituto Superiore di Sanità, 00161 Rome, Italy; luigina.ambrosio@iss.it (L.A.); angela.dimartino@iss.it (A.D.M.); annateresa.palamara@iss.it (A.T.P.); paola.stefanelli@iss.it (P.S.); 2 Department of Food Safety, Nutrition and Veterinary Public Health, Istituto Superiore di Sanità, 00161 Rome, Italy; arnold.knijn@iss.it (A.K.); luca.desabato@iss.it (L.D.S.); ilaria.dibartolo@iss.it (I.D.B.); stefano.morabito@iss.it (S.M.)

**Keywords:** genetic diversity, mutation, phylogenetic analysis, SARS-CoV-2, selective pressure

## Abstract

In this work, we studied the selective pressure and evolutionary analysis on the SARS-CoV-2 BF.7 and BQ.1.1 lineages circulating in Italy from July to December 2022. Two different datasets were constructed: the first comprised 694 SARS-CoV-2 BF.7 lineage sequences and the second comprised 734 BQ.1.1 sequences, available in the Italian COVID-19 Genomic (I-Co-Gen) platform and GISAID (last access date 15 December 2022). Alignments were performed with MAFFT v.7 under the Galaxy platform. The HYPHY software was used to study the selective pressure. Four positively selected sites (two in *nsp3* and two in the *spike*) were identified in the BF.7 dataset, and two (one in ORF8 and one in the spike gene) were identified in the BQ.1.1 dataset. Mutation analysis revealed that R408S and N440K are very common in the *spike* of the BF.7 genomes, as well as L452R among BQ.1.1. N1329D and Q180H in *nsp3* were found, respectively, at low and rare frequencies in BF.7, while I121L and I121T were found to be rare in ORF8 for BQ.1.1. The positively selected sites may have been driven by the selection for increased viral fitness, under circumstances of defined selective pressure, as well by host genetic factors.

## 1. Introduction

The SARS-CoV-2 virus evolved rapidly, with the emergence of new variants over time. The Omicron variant of SARS-CoV-2, first discovered in Botswana and South Africa on 11 November 2021, was subsequently identified worldwide. The World Health Organization designated Omicron as a variant of concern on 26 November 2021, as it became the leading variant [1].

The Omicron variant became dominant in Italy starting from January 2022 [2,3].

Omicron developed several sub-variants. In Italy, BA.5 showed a growing trend starting from July 2022, with a parallel decrease in the BA.2 sub-variant, reaching 81.7% of the sequences deposited on I-Co-Gen in the week 11–17 July 2022 [4] and a national prevalence of 90.8% at the end of August 2022 [5].

The BF.7 and BQ.1.1 sub-lineages of the Omicron variant BA.5 showed a growing trend in Italy starting from the end of November 2022 [6] in line with the international context [7,8].

The presence of the mutations K444T, N460K, and R346T in the spike of the sub-lineage BQ.1.1 and the R346T in the spike of BF.7 sub-lineage represents one of the main reasons to monitor these strains. In this regard, the receptor-binding domain (RBD) included position 346, whereas residue 658 is in close proximity to the S1/S2 cleavage [9].

Monitoring SARS-CoV-2 amino acid substitutions can help to explain viral behaviour and to detect potential alterations in transmissibility, infection severity, and immunity.

The amino-acid changes that result in reduced fitness are generally removed by negative selection, whereas changes that increase virus fitness are generally maintained by positive selection. The changes are considered neutral if they do not decrease or increase fitness.

In this work, we studied the selective pressure and evolutionary dynamics on SARS-CoV-2 BF.7 and BQ.1.1 lineages circulating in Italy from July to December 2022. The selective pressure allows us to estimate the nonsynonymous/synonymous rate (dN/dS, ω), considering a nonsynonymous rate higher than the synonymous as positive selection [10,11]. Since the selective pressure of SARS-CoV-2 BF.7 and BQ.1.1 lineages in Italy has not been investigated, this study might be useful to identify (i) the positive and negative selected sites and (ii) the recurrent and less frequent mutations.

## 2. Materials and Methods

### 2.1. Dataset and Sequence Alignment

All the high-quality (sequences containing <5% of ambiguous nucleotides N) SARS-CoV-2 genomes belonging to the BF.7 and BQ.1.1 lineages collected in Italy from July to December 2022, available in the national platform Italian COVID-19 Genomic I-Co-Gen (last access 15 December 2022) and GISAID [12,13], were downloaded.

The sequences (FASTA format) were downloaded from the I-Co-Gen platform and obtained through the open-source SARS-CoV-2 RECoVERY software v4.0 (developed by the ISS and available in the I-Co-Gen project), which automatically performs data quality control, the construction of genomes from NGS sequencing data (consensus) and other functions.

Therefore, two different datasets were investigated here. The first one is composed of 694 SARS-CoV-2 lineage BF.7 genomes; the second one included 734 lineage BQ.1.1 sequences. The GISAID Accession Numbers and the details of the sequences included in the first and second dataset were reported in Table S1.

In order to investigate the location of the Italian BF.7 and BQ.1.1 genomes in the phylogenetic trees with respect to those collected from other European countries, two additional datasets were created: the first one composed of a total of 2305 genomes (694 BF.7 Italian plus 1611 European) and the second one by 2459 genomes (734 BQ.1.1 Italian plus 1725 BQ.1.1 European). The lineage of the Italian sequences was assigned directly through the I-Co-Gen platform and the Pangolin 4.2 v1.17 software, and also confirmed uploading the sequences into the “Pangolin COVID-19 Lineage Assigner” online [14,15], last access 15 December 2022]. All the sequence alignments were obtained with the program MAFFT v.7 [16] under the Galaxy platform [17,18,19] and followed by manual editing through the Bioedit program [20].

### 2.2. Phylogenetic Analysis

The maximum likelihood phylogenetic trees on the two additional datasets were generated through IQ-TREE software using 1000 as the number of bootstrap replicates for branch support, SH-aLRT.

### 2.3. Selective Pressure Analysis

In this study, the following protein-coding gene sequence sub-sets (for both the first and second dataset) were created with the aim of investigating the SARS-CoV-2 amino acid substitutions, the evolutionary dynamics, and the positively and negatively selected sites: *nsp1*, *nsp2*, *nsp3*, *nsp4*, *3C-like proteinase* (*nsp5*), *nsp6*, *nsp7*, *nsp8*, *nsp9*, *nsp10*, *nsp11*, *nsp12*, *helicase* (*nsp13*), *3′-to-5′-exonuclease* (*nsp14*), *endoRNAse* (*nsp15*), *2′-O-ribosemethyltransferase* (*nsp16*), *S* (*surface* glycoprotein), *ORF3a*, *E*, *M*, N, *ORF6*, *ORF7a*, *ORF7b*, *ORF8,* and *ORF10*.

A positive diversifying selection occurred when ω > 1 (ω, rate of nonsynonymous substitutions to that of synonymous), while purifying selection was inferred for ω < 1 [21].

The models Fast Unconstrained Bayesian AppRoximation (FUBAR) [22], Fixed Effects Likelihood (FEL), and Single-Likelihood Ancestor Counting (SLAC) [23] were used in HYPHY software v 2.2.4 [23] to identify selection.

The method FUBAR infers the nonsynonymous (dN) and synonymous (dS) substitution rates on a per-site basis in large datasets, based on the assumption that a pervasive selection pressure is constant in the entire phylogeny [22], and with this method, improved robustness is obtained for large datasets [22]. The FEL model uses an ML approach to infer dN and dS substitution rates on a per-site basis [23], assuming that the selection for each site is constant along the phylogeny. The SLAC model uses a combination of maximum-likelihood and counting approaches to infer dN and dS substitution rates on a per-site basis. Additionally, in this case, the selection for each site is constant along with the phylogeny [23]. The SLAC uses ML to infer the most likely ancestral sequence at each node, with a Suzuki–Gojobori counting method to directly count the total number of nonsynonymous and synonymous changes at each site [23].

Only the sites found by at least two models under significant selection (FUBAR, posterior probability ≥ 0.9; FEL, *p* ≤ 0.05 and SLAC *p* ≤ 0.1) were reported. The amino acid positions of the sites were referred with respect to the protein products obtained from the SARS-CoV-2 reference sequence Wuhan-Hu-1 (Accession Number: NC_045512.2). The frequency of each amino acid substitutions in the positive selected sites was calculated in order to classify the mutations as very common (frequency ≥ 83.7%–84%), common (frequency ≥ 64.0%), low-frequency (between 2.1% and 21.0%) or rare (frequency ≤ 2.0%).

The prediction of the possible impact on protein stability of the amino acid substitutions found in the positively selected sites was investigated through I-Mutant 2.0 and PolyPhen-2 (Polymorphism Phenotyping v2) tools, as previously reported [24].

## 3. Results

### 3.1. Phylogenetic Analysis

The BF.7 and BQ.1.1 Italian genomes appeared in relation to foreign European sequences, but, beyond a general intermixing in the tree, several internal supported subclades/subclusters composed only by Italian genomes were identified, indicating a certain genomic divergence.

### 3.2. Selective Pressure Analysis

Overall, the selective pressure analysis showed a variation in the SARS-CoV-2 protein-coding genes (Table S2).

The first dataset (lineage BF.7) revealed a total of four significant positively selected sites, two of which were localized in the *nsp3* and two in the *spike*. The two sites (408, 440) identified in the *spike* of the lineage BF.7 dataset were located inside the RBD portion. Evidence of supported negative selection in the first dataset was detected for 29 sites, 5 of which (17.2%) were localized in *nsp3*, 5 (17.2%) in *nsp 13*, 4 (14.0%) in the *spike*, 3 (10.3%) in *nsp14*, 2 (6.9%) in *nsp5*, 2 (7.0%) in *nsp16*, 2 (7.0%) in N, and 1 (3.4%) in *nsp2*, *nsp4*, *nsp6*, *nsp10*, *nsp12*, *and ORF7a*. No positively or negatively selected sites were identified in *nsp1*, *nsp7*, *nsp8*, *nsp9*, *nsp11*, *nsp15*, *ORF3a*, *E*, *M*, *ORF6*, *ORF7b*, *ORF8*, *ORF10* (Table S2).

The second dataset (SARS-CoV-2 lineage BQ.1.1) was characterized by two statistically significant positively selected sites, one in *ORF8* and the other in the *spike* (Table S2). The positively selected site identified in the *spike* (lineage BQ.1.1 dataset) was located inside the RBD.

Negative selection for the second dataset was detected in 15 sites, 3 (20%) of which were localized in *nsp3*; 3 (20%) in *nsp15*; 2 (13.3%) in *nsp4*, *nsp16,* and *spike*; and 1 (6.7%) in *nsp1*, *nsp9,* and *ORF3a*. No positively or negatively selected sites were identified in *nsp2*, *nsp5*, *nsp6*, *nsp7*, *nsp8*, *nsp10*, *nsp11*, *nsp12*, *nsp13*, *nsp14*, *E*, *M*, *N*, *ORF6*, *ORF7a*, or *ORF7b*, *ORF10* (Table S2).

### 3.3. Frequency of the Amino Acid Substitutions Harbored by the Italian SARS-CoV-2 Genomes and the Prediction of the Possible Impact of the Amino Acid Substitutions

The positively selected sites were investigated to track the frequency of each amino acid replacement in our datasets (Table 1a,b).

The amino acid substitution N1329D in *nsp3* was identified rarely; the amino acid substitution Q180H in *nsp3* was identified at low frequency and the R408S, N440K) in the *spike* were detected as very common in the Italian SARS-CoV-2 BF.7 genomes (Table 1 panel a).

Meanwhile, Table 1 panel b indicates one substitution as very common (L452R in the spike) and two as rare (I121L, I121T in ORF8) in the Italian BQ.1.1 lineage genomes (second dataset).

**Figure 1:**
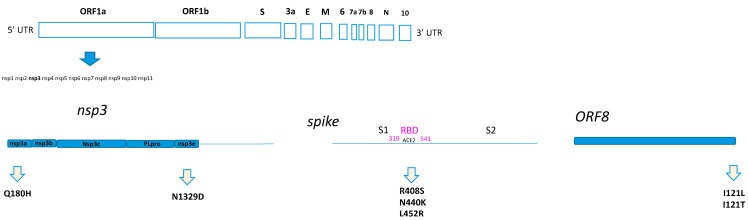
Home-made schematic diagram relating to the location of the amino acid substitutions on the SARS-CoV-2 proteins.

In particular, Q180H was located at the N-terminal of NSP3, in the nsp3a portion. Meanwhile, the N1329D was located in the nsp3e portion. All the amino acid substitutions identified as positively selected sites in the spike were located inside the RBD (Figure 1). Finally, the I121L and I121T were found in ORF8, which is one of the so-called accessory proteins, composed of 121-amino acids (Figure 1).

Table 2 shows that 75% of the amino acid replacements identified as positively selected sites in the first dataset were predicted to decrease, while 25% were predicted to increase the stability of the proteins. All the amino acid replacements identified as positively selected sites for the second dataset (100%) were predicted to decrease the stability of the reported proteins (Table 2). Only two amino acid replacements (one in the first and the other in the second dataset) were predicted by PolyPhen-2 as probably or possibly damaging the protein structure (Table 2, score > 0.92). The remaining mutations were predicted with a benign effect on the protein structure (Table 2).

## 4. Discussion

The SARS-CoV-2 virus evolved rapidly with the emergence of new variants over time.

Tracking the evolution of SARS-CoV-2 offers the opportunity to understand the viral genetic diversity, potentially predicting possible future evolutionary trajectories of the virus and offering routes for prevention and treatment.

Omicron’s BF.7 and BQ.1 variants have circulated widely in different parts of the world [25,26] and also in Italy [6]. Both revealed increased resistance to neutralization antibodies [25] and increased ACE2 binding affinity, infectivity, and fusogenicity [26].

This study provides a genomic and evolutionary analysis of SARS-CoV-2 BF.7 and BQ.1.1 lineages circulating in Italy from July to December 2022 with the aim to understand their fitness landscape, the selective pressure, and the amino acid changes with a selective advantage. Although these sublineages are actually bypassed by others, which emerged later, the selective pressure, the mutational profile, and the genetic diversity of the SARS-CoV-2 BF.7 and BQ.1.1 Italian genomes can contribute to expanding the data available from a large dataset, particularly on the amino acid substitutions identified. The positively selected sites identified here suggest that they may have been driven by selection for increased viral fitness under defined selective pressure circumstances, as well as by possible host genetic factors. Notably, the mutations R408S and N440K were identified in a higher frequency in the spike of the Italian BF.7 dataset. The accumulation of mutations can occur faster if some of them are considered advantageous. Some studies showed that the mutation R408S can lead to the escape of the antibodies and can induce smaller binding free energy changes in RBD-human ACE2 complexes [27], reducing the efficacy of many antibodies [28]. The N440K mutation has also been reported to escape antibody neutralization, which could increase infection risk [29] while also showing increased infectious fitness [30].

As regards the data on *nsp3*, the Q180H and N1329D mutations were identified at a low frequency (2.59%) in Italian genomes of the BF.7 lineage. In particular, literature data showed that the frequency of the Q180H mutation ranged from 0.05% to 1.18% in SARS-CoV-2 genomes collected in Africa, Asia, Australia, Europe, North America, and South America [31,32]. The same authors identified the mutation Q180H with a significant negative correlation with a case fatality ratio. The frequency of Q180H (*nsp3)* in the BF.7 lineage in specific European countries showed values ranging from 0.11% (France), 0.32% (Spain), 0.43% (Austria), 1.76% (Slovenia), and 1.67% (Switzerland), according to GISAID data [12, 13, last accessed 25 March 2024]. Local genomic evolution could partly explain our data.

The mutation N1329D identified in the *nsp3* of the Italian BF.7 dataset in this study, if compared to BF.7 genomes from GISAID [12, 13, last accessed 25 March 2024], was reported in some Italian genomes and only in one foreign BF.7 genome collected in Austria (EPI_ISL_16377903). This mutation should be better evaluated in future studies since it falls within the nsp3e. Our data reinforce the concept that the substitutions that fall within the non-structural proteins, together with their prevalences, need to be monitored, in particular those occurring inside the *nsp3e*, which play an important role in the virus RNA synthesis and replication, suggesting that they may be useful targets for anti-viral drug discovery or therapeutic strategies [33].

In the SARS-CoV-2 BQ.1.1 dataset, the L452R (*spike*), was identified as a very common amino acid substitution, in line with what also occurred in other BQ.1.1 European genomes (data from GISAID). The L452R plays a key role, as it has been reported to increase SARS-CoV-2 fusogenicity and infectivity [34].

Even though I121L and I121T (ORF8, BQ.1.1 dataset) were identified at a low frequency in this study, they have to be closely monitored because they are localized in *ORF8*, a protein that has several important functions within SARS-CoV-2 pathogenesis [35].

Finally, to better understand the functional characteristics of the nonsynonymous mutations identified in the positively selected sites, the changes in the proteins were evaluated using PolyPhen-2, while I-Mutant 2.0 was used to evaluate their structural stability.

Some amino acid changes are expected to have a possible impact on the structure and stability of proteins, and for all these reasons, the need to be closely monitored. The evolution of SARS-CoV-2 will depend on the intensity and rate of fixation of further evolutionary changes and on the rate of emergence of distinct lineages. For this reason it is important to continue to monitor the selective pressure to identify relevant mutational “hot spots” and potentially useful to roll out and update vaccines and/or therapies.

Before drawing conclusions, it is worth mentioning the possible limitations of this study. In particular, the model depends on the availability of SARS-CoV-2 genomes and can to some extent influence the models used for the analysis.

## 5. Conclusions

In conclusion, our study provides an overview of the selective pressure profile in a dataset of SARS-CoV-2 BF.7 and BQ.1.1 lineages identified in Italy, highlighting how specific key mutations can become fixed in this viral population and the possible impact on the stability of the proteins. Furthermore, a specific key mutation (N1329D—*nsp3*) needs to be better evaluated in future studies to better clarify its role and impact.

## Figures and Tables

**Table 1:** Panel (**a**): Frequency of amino acid substitutions harbored by the Italian BF.7 lineage genomes (first dataset, *n* = 694) and detected as positively selected sites. Panel (**b**): Frequency of amino acid substitutions harbored by the Italian BQ.1.1 lineage genomes (second dataset, *n* = 734) and detected as positively selected sites.

**(a)**		
**Amino acid substitution**	**Percentage**	**Target**
Q180H	2.59%	*nsp3*
N1329D	1.58%	*nsp3*
R408S	94.81%	*spike*
N440K	93.08%	*spike*
**(b)**		
**Amino acid substitution**	**Percentage**	**Target**
I121L	0.14%	*ORF8*
I121T	0.14%	*ORF8*
L452R	84.00%	*spike*

**Table 2:** Results of the prediction obtained through I-Mutant 2.0 and PolyPhen-2.

			PolyPhen-2		I-Mutant 2.0	
Amino Acid Substitution	Dataset	Target	Prediction	Score	Stability	DDG
Q180H	first	nsp3	Benign	0.306	Increase	0.21
N1329D	first	nsp3	Benign	0.052	Decrease	−0.42
R408S	first	spike	Probably Damaging	1	Decrease	−2.2
N440K	first	spike	Benign	0.003	Decrease	−0.86
I121L	second	ORF8	Benign	0.01	Decrease	−0.44
I121T	second	ORF8	Possibly Damaging	0.925	Decrease	−2.66
L452R	second	spike	Benign	0.017	Decrease	−1.4

## Data Availability

The sequences used in this study were available in GISAID (https://gisaid.org/ accessed on 15 December 2022).

## Supplementary Materials

The following supporting information can be downloaded at https://www.mdpi.com/article/10.3390/microorganisms12050908/s1. Table S1: (a) GISAID Accession Numbers of SARS-CoV-2 genomes belonging to BF.7 lineage (first dataset); (b) GISAID Accession Numbers of SARS-CoV-2 genomes belonging to BQ.1.1 lineage (second dataset). Table S2: Selective pressure analysis on SARS-CoV-2 lineages BF.7 and BQ.1.1.
