# Supplementary material for: Selective Pressure and Evolution of SARS-CoV-2 Lineages BF.7 and BQ.1.1 Circulating in Italy from July to December 2022
Source: Microorganisms. 2024 Apr 30;12(5):908. doi: 10.3390/microorganisms12050908 (PMC11124320; doi:10.3390/microorganisms12050908)
Supplement: Supplementary file 1 [file microorganisms-12-00908-s001.zip › Table S1 REVISED.pdf]

Table S1. a) GISAID Accession Numbers of SARS-CoV-2 genomes belonging to BF.7 lineage (first dataset)

EPI_ISL_13740570	EPI_ISL_14995693	EPI_ISL_15252685	EPI_ISL_15368172
EPI_ISL_14200641	EPI_ISL_14995709	EPI_ISL_15252671	EPI_ISL_15368157
EPI_ISL_14570455	EPI_ISL_14995711	EPI_ISL_15252651	EPI_ISL_15368139
EPI_ISL_14386927	EPI_ISL_14995688	EPI_ISL_15283109	EPI_ISL_15368111
EPI_ISL_14386715	EPI_ISL_15019992	EPI_ISL_15282944	EPI_ISL_15368112
EPI_ISL_14357120	EPI_ISL_15019986	EPI_ISL_15282847	EPI_ISL_15343658
EPI_ISL_14390379	EPI_ISL_15019984	EPI_ISL_15282752	EPI_ISL_15343657
EPI_ISL_14390365	EPI_ISL_15019976	EPI_ISL_15282704	EPI_ISL_15329656
EPI_ISL_14390350	EPI_ISL_15233268	EPI_ISL_15282671	EPI_ISL_15330016
EPI_ISL_14390108	EPI_ISL_15247599	EPI_ISL_15282610	EPI_ISL_15505227
EPI_ISL_14393480	EPI_ISL_15022816	EPI_ISL_15284304	EPI_ISL_15505211
EPI_ISL_14393511	EPI_ISL_15056590	EPI_ISL_15383386	EPI_ISL_15333945
EPI_ISL_14474711	EPI_ISL_15069377	EPI_ISL_15383370	EPI_ISL_15333948
EPI_ISL_14493888	EPI_ISL_15069373	EPI_ISL_15292630	EPI_ISL_15388728
EPI_ISL_14493887	EPI_ISL_15083840	EPI_ISL_15292647	EPI_ISL_15350185
EPI_ISL_14542230	EPI_ISL_15083845	EPI_ISL_15292648	EPI_ISL_15350178
EPI_ISL_14542091	EPI_ISL_15084123	EPI_ISL_15292653	EPI_ISL_15350173
EPI_ISL_14549724	EPI_ISL_15084069	EPI_ISL_15292654	EPI_ISL_15350151
EPI_ISL_14932009	EPI_ISL_15099090	EPI_ISL_15292660	EPI_ISL_15350138
EPI_ISL_14855045	EPI_ISL_15099084	EPI_ISL_15292662	EPI_ISL_15350119
EPI_ISL_14798120	EPI_ISL_15102714	EPI_ISL_15292663	EPI_ISL_15350116
EPI_ISL_14786130	EPI_ISL_15102671	EPI_ISL_15292666	EPI_ISL_15350118
EPI_ISL_14815390	EPI_ISL_15247533	EPI_ISL_15292671	EPI_ISL_15351976
EPI_ISL_14887905	EPI_ISL_15247525	EPI_ISL_15303260	EPI_ISL_15362987
EPI_ISL_14913920	EPI_ISL_15112675	EPI_ISL_15512690	EPI_ISL_15350917
EPI_ISL_14913915	EPI_ISL_15112661	EPI_ISL_15512697	EPI_ISL_15350923
EPI_ISL_14980662	EPI_ISL_15154250	EPI_ISL_15512706	EPI_ISL_15350924
EPI_ISL_14924698	EPI_ISL_15157684	EPI_ISL_15512689	EPI_ISL_15350925
EPI_ISL_14922309	EPI_ISL_15188070	EPI_ISL_15512711	EPI_ISL_15362818
EPI_ISL_14922312	EPI_ISL_15188071	EPI_ISL_15512707	EPI_ISL_15362806
EPI_ISL_14922313	EPI_ISL_15188079	EPI_ISL_15512703	EPI_ISL_15362803
EPI_ISL_14922317	EPI_ISL_15247406	EPI_ISL_15388789	EPI_ISL_15362788
EPI_ISL_15363750	EPI_ISL_15247400	EPI_ISL_15388761	EPI_ISL_15362764
EPI_ISL_15363741	EPI_ISL_15192673	EPI_ISL_15306898	EPI_ISL_15362711
EPI_ISL_15363732	EPI_ISL_15192680	EPI_ISL_15315933	EPI_ISL_15363549
EPI_ISL_15363728	EPI_ISL_15192683	EPI_ISL_15315930	EPI_ISL_15363527
EPI_ISL_14971404	EPI_ISL_15192701	EPI_ISL_15315924	EPI_ISL_15363522
EPI_ISL_14971348	EPI_ISL_15192706	EPI_ISL_15368313	EPI_ISL_15364995
EPI_ISL_14971326	EPI_ISL_15208731	EPI_ISL_15368296	EPI_ISL_15364975
EPI_ISL_14992409	EPI_ISL_15208725	EPI_ISL_15368298	EPI_ISL_15364968
EPI_ISL_14992396	EPI_ISL_15220979	EPI_ISL_15368201	EPI_ISL_15364953
EPI_ISL_14991655	EPI_ISL_15247339	EPI_ISL_15368182	EPI_ISL_15364947
EPI_ISL_14994383	EPI_ISL_15247337	EPI_ISL_15368161	EPI_ISL_15364895

EPI_ISL_15364883	EPI_ISL_15436118	EPI_ISL_15493874	EPI_ISL_15636850
EPI_ISL_15364885	EPI_ISL_15436123	EPI_ISL_15494288	EPI_ISL_15754190
EPI_ISL_15364882	EPI_ISL_15436144	EPI_ISL_15494280	EPI_ISL_15656064
EPI_ISL_15364881	EPI_ISL_15436147	EPI_ISL_15630403	EPI_ISL_15656062
EPI_ISL_15364880	EPI_ISL_15436148	EPI_ISL_15630393	EPI_ISL_15656060
EPI_ISL_15364877	EPI_ISL_15436149	EPI_ISL_15630387	EPI_ISL_15656048
EPI_ISL_15364878	EPI_ISL_15436152	EPI_ISL_15537461	EPI_ISL_15656050
EPI_ISL_15364879	EPI_ISL_15436155	EPI_ISL_15537183	EPI_ISL_15656042
EPI_ISL_15364876	EPI_ISL_15195486	EPI_ISL_15537190	EPI_ISL_15656039
EPI_ISL_15364875	EPI_ISL_15427026	EPI_ISL_15537197	EPI_ISL_15656038
EPI_ISL_15364874	EPI_ISL_15427047	EPI_ISL_15537201	EPI_ISL_15656036
EPI_ISL_15364868	EPI_ISL_15427067	EPI_ISL_15537203	EPI_ISL_15656034
EPI_ISL_15364867	EPI_ISL_15427144	EPI_ISL_15537208	EPI_ISL_15661812
EPI_ISL_15364866	EPI_ISL_15427273	EPI_ISL_15537209	EPI_ISL_15661818
EPI_ISL_15365032	EPI_ISL_15427274	EPI_ISL_15537211	EPI_ISL_15661822
EPI_ISL_15365029	EPI_ISL_15427299	EPI_ISL_15537215	EPI_ISL_15661824
EPI_ISL_15365015	EPI_ISL_15427300	EPI_ISL_15541887	EPI_ISL_15661834
EPI_ISL_15366500	EPI_ISL_15470001	EPI_ISL_15541892	EPI_ISL_15661842
EPI_ISL_15367171	EPI_ISL_15470003	EPI_ISL_15541886	EPI_ISL_15661846
EPI_ISL_15364802	EPI_ISL_15470004	EPI_ISL_15541884	EPI_ISL_15661849
EPI_ISL_15364804	EPI_ISL_15509781	EPI_ISL_15541880	EPI_ISL_15661852
EPI_ISL_15364808	EPI_ISL_15509776	EPI_ISL_15541877	EPI_ISL_15661855
EPI_ISL_15364823	EPI_ISL_15479411	EPI_ISL_15541760	EPI_ISL_15665402
EPI_ISL_15367208	EPI_ISL_15479408	EPI_ISL_15541756	EPI_ISL_15665396
EPI_ISL_15367197	EPI_ISL_15479402	EPI_ISL_15630352	EPI_ISL_15665395
EPI_ISL_15368244	EPI_ISL_15482145	EPI_ISL_15630349	EPI_ISL_15665394
EPI_ISL_15368229	EPI_ISL_15482143	EPI_ISL_15630343	EPI_ISL_15668556
EPI_ISL_15368216	EPI_ISL_15482135	EPI_ISL_15630337	EPI_ISL_15720520
EPI_ISL_15388702	EPI_ISL_15482133	EPI_ISL_15630331	EPI_ISL_15720518
EPI_ISL_15388693	EPI_ISL_15482130	EPI_ISL_15630332	EPI_ISL_15720509
EPI_ISL_15418159	EPI_ISL_15482119	EPI_ISL_15612030	EPI_ISL_15682647
EPI_ISL_15418148	EPI_ISL_15482111	EPI_ISL_15611867	EPI_ISL_15720500
EPI_ISL_15418138	EPI_ISL_15482108	EPI_ISL_15611860	EPI_ISL_15720496
EPI_ISL_15422156	EPI_ISL_15581746	EPI_ISL_15545075	EPI_ISL_15720490
EPI_ISL_15422133	EPI_ISL_15581744	EPI_ISL_15545090	EPI_ISL_15720473
EPI_ISL_15422132	EPI_ISL_15581733	EPI_ISL_15545100	EPI_ISL_15697161
EPI_ISL_15422128	EPI_ISL_15581728	EPI_ISL_15581166	EPI_ISL_15697159
EPI_ISL_15422134	EPI_ISL_15581718	EPI_ISL_15581158	EPI_ISL_15697156
EPI_ISL_15422127	EPI_ISL_15493895	EPI_ISL_15630317	EPI_ISL_15697150
EPI_ISL_15422129	EPI_ISL_15493891	EPI_ISL_15630326	EPI_ISL_15697143
EPI_ISL_15422119	EPI_ISL_15493884	EPI_ISL_15630305	EPI_ISL_15697140
EPI_ISL_15509829	EPI_ISL_15493879	EPI_ISL_15636856	EPI_ISL_15697139
EPI_ISL_15509814	EPI_ISL_15493875	EPI_ISL_15636855	EPI_ISL_15697129

EPI_ISL_15697133	EPI_ISL_15769846	EPI_ISL_15790718	EPI_ISL_15855217
EPI_ISL_15697126	EPI_ISL_15769709	EPI_ISL_15790719	EPI_ISL_15997747
EPI_ISL_15722049	EPI_ISL_15769705	EPI_ISL_15790715	EPI_ISL_15997741
EPI_ISL_15731118	EPI_ISL_15769695	EPI_ISL_15803226	EPI_ISL_15997743
EPI_ISL_15731115	EPI_ISL_15769692	EPI_ISL_15803218	EPI_ISL_15997722
EPI_ISL_15731108	EPI_ISL_15790617	EPI_ISL_15803217	EPI_ISL_15877711
EPI_ISL_15731099	EPI_ISL_15971238	EPI_ISL_15803206	EPI_ISL_15877712
EPI_ISL_15743861	EPI_ISL_15971242	EPI_ISL_15803202	EPI_ISL_15898883
EPI_ISL_15754175	EPI_ISL_15800507	EPI_ISL_15833151	EPI_ISL_15898857
EPI_ISL_15754155	EPI_ISL_15800491	EPI_ISL_15833125	EPI_ISL_15898850
EPI_ISL_15750047	EPI_ISL_15800489	EPI_ISL_15801506	EPI_ISL_15898831
EPI_ISL_15750048	EPI_ISL_15800480	EPI_ISL_15801507	EPI_ISL_15898796
EPI_ISL_15750053	EPI_ISL_15800476	EPI_ISL_15992133	EPI_ISL_15898794
EPI_ISL_15750057	EPI_ISL_15970966	EPI_ISL_15808973	EPI_ISL_15898793
EPI_ISL_15750058	EPI_ISL_15970971	EPI_ISL_15808975	EPI_ISL_15898778
EPI_ISL_15750059	EPI_ISL_15800446	EPI_ISL_15808985	EPI_ISL_15898779
EPI_ISL_15750066	EPI_ISL_15800424	EPI_ISL_15808987	EPI_ISL_15898785
EPI_ISL_15750072	EPI_ISL_15800422	EPI_ISL_15808989	EPI_ISL_15898782
EPI_ISL_15750079	EPI_ISL_15776882	EPI_ISL_15808990	EPI_ISL_15898780
EPI_ISL_15750087	EPI_ISL_15776880	EPI_ISL_15808992	EPI_ISL_15898770
EPI_ISL_15745056	EPI_ISL_15776874	EPI_ISL_15808994	EPI_ISL_15898776
EPI_ISL_15745057	EPI_ISL_15776868	EPI_ISL_15809010	EPI_ISL_15898756
EPI_ISL_15745064	EPI_ISL_15778240	EPI_ISL_15809011	EPI_ISL_15898747
EPI_ISL_15745074	EPI_ISL_15778238	EPI_ISL_15809012	EPI_ISL_15898735
EPI_ISL_15759019	EPI_ISL_15778241	EPI_ISL_15809016	EPI_ISL_15898739
EPI_ISL_15759003	EPI_ISL_15781885	EPI_ISL_15814144	EPI_ISL_15898742
EPI_ISL_15758991	EPI_ISL_15781890	EPI_ISL_15814152	EPI_ISL_15898710
EPI_ISL_15758988	EPI_ISL_15781891	EPI_ISL_15814160	EPI_ISL_15898672
EPI_ISL_15760338	EPI_ISL_15781887	EPI_ISL_15814161	EPI_ISL_15898676
EPI_ISL_15760263	EPI_ISL_15781869	EPI_ISL_15814167	EPI_ISL_15898654
EPI_ISL_15760252	EPI_ISL_15781871	EPI_ISL_16055757	EPI_ISL_15898646
EPI_ISL_15760248	EPI_ISL_15796094	EPI_ISL_16055752	EPI_ISL_15898633
EPI_ISL_15762576	EPI_ISL_15796093	EPI_ISL_15837584	EPI_ISL_15898611
EPI_ISL_15762574	EPI_ISL_15796095	EPI_ISL_15837585	EPI_ISL_15898617
EPI_ISL_15792059	EPI_ISL_15796092	EPI_ISL_15837576	EPI_ISL_15898619
EPI_ISL_15762566	EPI_ISL_15796088	EPI_ISL_15837579	EPI_ISL_15898574
EPI_ISL_15762525	EPI_ISL_15796066	EPI_ISL_15837568	EPI_ISL_15898567
EPI_ISL_15762524	EPI_ISL_15796060	EPI_ISL_15837563	EPI_ISL_15898555
EPI_ISL_16055802	EPI_ISL_15796056	EPI_ISL_15837556	EPI_ISL_15898542
EPI_ISL_16055794	EPI_ISL_15796055	EPI_ISL_15837546	EPI_ISL_15895369
EPI_ISL_16055785	EPI_ISL_15790747	EPI_ISL_15837540	EPI_ISL_15895348
EPI_ISL_16055771	EPI_ISL_15790745	EPI_ISL_15837845	EPI_ISL_15997980
EPI_ISL_16055768	EPI_ISL_15790748	EPI_ISL_15855220	EPI_ISL_15997972

EPI_ISL_15997971	EPI_ISL_15950003	EPI_ISL_16017244	EPI_ISL_16093710
EPI_ISL_15997967	EPI_ISL_15949994	EPI_ISL_16017242	EPI_ISL_16093703
EPI_ISL_15997964	EPI_ISL_15955384	EPI_ISL_16017240	EPI_ISL_16093702
EPI_ISL_15997962	EPI_ISL_15955390	EPI_ISL_16017228	EPI_ISL_16077168
EPI_ISL_15897929	EPI_ISL_15955385	EPI_ISL_16017229	EPI_ISL_16077163
EPI_ISL_15908771	EPI_ISL_15955373	EPI_ISL_16017232	EPI_ISL_16077164
EPI_ISL_15908777	EPI_ISL_15957771	EPI_ISL_16017230	EPI_ISL_16077170
EPI_ISL_15908781	EPI_ISL_15957756	EPI_ISL_16018033	EPI_ISL_16077139
EPI_ISL_15908789	EPI_ISL_15964540	EPI_ISL_16018029	EPI_ISL_16080101
EPI_ISL_15908791	EPI_ISL_15964541	EPI_ISL_16018574	EPI_ISL_16080096
EPI_ISL_15908806	EPI_ISL_15964522	EPI_ISL_16018572	EPI_ISL_16080093
EPI_ISL_15908807	EPI_ISL_15998901	EPI_ISL_16018565	EPI_ISL_16080091
EPI_ISL_15908812	EPI_ISL_15973010	EPI_ISL_16018004	EPI_ISL_16093931
EPI_ISL_15908813	EPI_ISL_15983068	EPI_ISL_16017513	EPI_ISL_16093929
EPI_ISL_15910010	EPI_ISL_15983071	EPI_ISL_16017510	EPI_ISL_16122499
EPI_ISL_15912355	EPI_ISL_15983076	EPI_ISL_16020315	EPI_ISL_16122486
EPI_ISL_15912350	EPI_ISL_15983077	EPI_ISL_16020306	EPI_ISL_16122477
EPI_ISL_15912348	EPI_ISL_15983078	EPI_ISL_16020309	EPI_ISL_16122455
EPI_ISL_15912969	EPI_ISL_15983079	EPI_ISL_16020291	EPI_ISL_16122423
EPI_ISL_15916609	EPI_ISL_15983100	EPI_ISL_16020286	EPI_ISL_16122418
EPI_ISL_15916588	EPI_ISL_15983105	EPI_ISL_16020289	EPI_ISL_16122412
EPI_ISL_15997949	EPI_ISL_15983790	EPI_ISL_16020633	EPI_ISL_16122417
EPI_ISL_15997958	EPI_ISL_15983785	EPI_ISL_16036634	EPI_ISL_16122413
EPI_ISL_15997940	EPI_ISL_15983779	EPI_ISL_16036631	EPI_ISL_16122383
EPI_ISL_15938095	EPI_ISL_15983774	EPI_ISL_16052030	EPI_ISL_16122382
EPI_ISL_15938085	EPI_ISL_15983758	EPI_ISL_16052031	EPI_ISL_16122375
EPI_ISL_15938077	EPI_ISL_15983753	EPI_ISL_16052034	EPI_ISL_16122340
EPI_ISL_15938078	EPI_ISL_15983738	EPI_ISL_16052040	EPI_ISL_16122337
EPI_ISL_15938063	EPI_ISL_15983727	EPI_ISL_16052042	EPI_ISL_16122324
EPI_ISL_15938064	EPI_ISL_15983726	EPI_ISL_16052044	EPI_ISL_16122330
EPI_ISL_15938060	EPI_ISL_15983722	EPI_ISL_16052046	EPI_ISL_16122321
EPI_ISL_15938058	EPI_ISL_15999321	EPI_ISL_16052053	EPI_ISL_16122304
EPI_ISL_15938045	EPI_ISL_15999318	EPI_ISL_16052057	EPI_ISL_16122307
EPI_ISL_15938043	EPI_ISL_16018036	EPI_ISL_16052058	EPI_ISL_16122308
EPI_ISL_15938034	EPI_ISL_16004457	EPI_ISL_16052059	EPI_ISL_16122298
EPI_ISL_15998888	EPI_ISL_16018035	EPI_ISL_16052062	EPI_ISL_16122275
EPI_ISL_15998889	EPI_ISL_16008315	EPI_ISL_16052066	EPI_ISL_16122267
EPI_ISL_15998873	EPI_ISL_16008310	EPI_ISL_16055399	EPI_ISL_16122274
EPI_ISL_15941864	EPI_ISL_16017263	EPI_ISL_16093759	EPI_ISL_16122266
EPI_ISL_15941853	EPI_ISL_16017256	EPI_ISL_16093754	EPI_ISL_16122242
EPI_ISL_15941843	EPI_ISL_16017250	EPI_ISL_16093743	EPI_ISL_16122245
EPI_ISL_16055716	EPI_ISL_16017249	EPI_ISL_16093737	EPI_ISL_16122232
EPI_ISL_16055712	EPI_ISL_16017246	EPI_ISL_16093713	EPI_ISL_16122230

EPI_ISL_16122223
EPI_ISL_16122213
EPI_ISL_16122209
EPI_ISL_16122207
EPI_ISL_16122164
EPI_ISL_16122169

Table S1. b) GISAID Accession Numbers of SARS-CoV-2 genomes belonging to BQ.1.1 lineage (second dataset)

EPI_ISL_15247559	EPI_ISL_15479417	EPI_ISL_15656057	EPI_ISL_15743869
EPI_ISL_15102759	EPI_ISL_15479380	EPI_ISL_15661809	EPI_ISL_15743862
EPI_ISL_15102635	EPI_ISL_15479369	EPI_ISL_15661828	EPI_ISL_15754178
EPI_ISL_15192446	EPI_ISL_15482165	EPI_ISL_15661830	EPI_ISL_15754177
EPI_ISL_15192442	EPI_ISL_15482148	EPI_ISL_15661838	EPI_ISL_15754176
EPI_ISL_15512708	EPI_ISL_15482121	EPI_ISL_15661839	EPI_ISL_15754168
EPI_ISL_15306908	EPI_ISL_15482114	EPI_ISL_15661841	EPI_ISL_15754162
EPI_ISL_15306905	EPI_ISL_15494278	EPI_ISL_15661847	EPI_ISL_15754159
EPI_ISL_15334115	EPI_ISL_15630397	EPI_ISL_15665404	EPI_ISL_15754163
EPI_ISL_15388731	EPI_ISL_15630398	EPI_ISL_15665397	EPI_ISL_15754157
EPI_ISL_15351975	EPI_ISL_15630401	EPI_ISL_15668582	EPI_ISL_15754150
EPI_ISL_15350927	EPI_ISL_15630395	EPI_ISL_15682923	EPI_ISL_15754148
EPI_ISL_15350930	EPI_ISL_15630391	EPI_ISL_15682921	EPI_ISL_15754152
EPI_ISL_15350932	EPI_ISL_15630386	EPI_ISL_15682920	EPI_ISL_15754151
EPI_ISL_15362790	EPI_ISL_15630380	EPI_ISL_15682662	EPI_ISL_15750042
EPI_ISL_15362744	EPI_ISL_15630384	EPI_ISL_15682639	EPI_ISL_15750049
EPI_ISL_15364990	EPI_ISL_15630359	EPI_ISL_15682913	EPI_ISL_15750069
EPI_ISL_15364370	EPI_ISL_15537457	EPI_ISL_15720498	EPI_ISL_15750071
EPI_ISL_15364365	EPI_ISL_15537192	EPI_ISL_15720494	EPI_ISL_15750074
EPI_ISL_15364361	EPI_ISL_15537195	EPI_ISL_15720499	EPI_ISL_15750077
EPI_ISL_15364916	EPI_ISL_15537212	EPI_ISL_15720487	EPI_ISL_15750083
EPI_ISL_15366524	EPI_ISL_15537216	EPI_ISL_15720485	EPI_ISL_15750084
EPI_ISL_15367187	EPI_ISL_15537218	EPI_ISL_15720483	EPI_ISL_15750085
EPI_ISL_15367196	EPI_ISL_15541769	EPI_ISL_15720484	EPI_ISL_15757813
EPI_ISL_15378463	EPI_ISL_15541765	EPI_ISL_15720472	EPI_ISL_15757807
EPI_ISL_15378462	EPI_ISL_15630348	EPI_ISL_15697125	EPI_ISL_15745049
EPI_ISL_15378461	EPI_ISL_15630334	EPI_ISL_15697119	EPI_ISL_15759009
EPI_ISL_15378460	EPI_ISL_15612028	EPI_ISL_15697111	EPI_ISL_15758987
EPI_ISL_15378459	EPI_ISL_15612026	EPI_ISL_15722060	EPI_ISL_15792068
EPI_ISL_15388706	EPI_ISL_15612017	EPI_ISL_15725982	EPI_ISL_15760337
EPI_ISL_15418142	EPI_ISL_15611988	EPI_ISL_15725981	EPI_ISL_15760331
EPI_ISL_15422144	EPI_ISL_15545088	EPI_ISL_15725954	EPI_ISL_15760329
EPI_ISL_15422135	EPI_ISL_15545097	EPI_ISL_15743216	EPI_ISL_15760327
EPI_ISL_15509833	EPI_ISL_15545104	EPI_ISL_15743207	EPI_ISL_15760322
EPI_ISL_15509816	EPI_ISL_15573043	EPI_ISL_15743195	EPI_ISL_15760265

EPI_ISL_15509810	EPI_ISL_15573037	EPI_ISL_15743185	EPI_ISL_15760268
EPI_ISL_15464205	EPI_ISL_15573031	EPI_ISL_15743189	EPI_ISL_15760259
EPI_ISL_15464201	EPI_ISL_15630319	EPI_ISL_15743179	EPI_ISL_15760257
EPI_ISL_15464193	EPI_ISL_15630318	EPI_ISL_15743180	EPI_ISL_15760255
EPI_ISL_15436142	EPI_ISL_15630301	EPI_ISL_15743183	EPI_ISL_15792067
EPI_ISL_15436153	EPI_ISL_15612391	EPI_ISL_15731081	EPI_ISL_15762571
EPI_ISL_15470005	EPI_ISL_15612386	EPI_ISL_15731079	EPI_ISL_15762570
EPI_ISL_15469996	EPI_ISL_15612375	EPI_ISL_15731077	EPI_ISL_15792051
EPI_ISL_15469988	EPI_ISL_15754185	EPI_ISL_15731076	EPI_ISL_15792050
EPI_ISL_15472436	EPI_ISL_15754180	EPI_ISL_15731075	EPI_ISL_15762563
EPI_ISL_15509800	EPI_ISL_15656061	EPI_ISL_15743875	EPI_ISL_15762558
EPI_ISL_16055807	EPI_ISL_15803229	EPI_ISL_15814204	EPI_ISL_15898631
EPI_ISL_15763836	EPI_ISL_15803207	EPI_ISL_15814207	EPI_ISL_15898618
EPI_ISL_16055801	EPI_ISL_15803199	EPI_ISL_15837578	EPI_ISL_15898609
EPI_ISL_16055797	EPI_ISL_15803200	EPI_ISL_15837587	EPI_ISL_15898601
EPI_ISL_16055793	EPI_ISL_15803194	EPI_ISL_15837580	EPI_ISL_15898598
EPI_ISL_16055780	EPI_ISL_15803192	EPI_ISL_15837569	EPI_ISL_15898595
EPI_ISL_16055769	EPI_ISL_15803193	EPI_ISL_15837565	EPI_ISL_15898586
EPI_ISL_16055764	EPI_ISL_15833146	EPI_ISL_15837558	EPI_ISL_15898578
EPI_ISL_16055761	EPI_ISL_15833145	EPI_ISL_15837549	EPI_ISL_15898572
EPI_ISL_16055762	EPI_ISL_15833142	EPI_ISL_15837547	EPI_ISL_15898563
EPI_ISL_15769853	EPI_ISL_15833141	EPI_ISL_15837847	EPI_ISL_15898558
EPI_ISL_15769843	EPI_ISL_15833140	EPI_ISL_15837836	EPI_ISL_15898559
EPI_ISL_15769718	EPI_ISL_15833136	EPI_ISL_15837828	EPI_ISL_15898539
EPI_ISL_15769715	EPI_ISL_15833137	EPI_ISL_15855224	EPI_ISL_15895377
EPI_ISL_15769699	EPI_ISL_15833133	EPI_ISL_15855219	EPI_ISL_15895374
EPI_ISL_15769691	EPI_ISL_15833129	EPI_ISL_15997750	EPI_ISL_15895376
EPI_ISL_15769690	EPI_ISL_15801526	EPI_ISL_15997745	EPI_ISL_15895370
EPI_ISL_15769686	EPI_ISL_15801521	EPI_ISL_15997749	EPI_ISL_15895359
EPI_ISL_15800498	EPI_ISL_15801522	EPI_ISL_15997751	EPI_ISL_15895351
EPI_ISL_15800474	EPI_ISL_15801525	EPI_ISL_15997738	EPI_ISL_15895350
EPI_ISL_15971078	EPI_ISL_15801510	EPI_ISL_15997737	EPI_ISL_15895347
EPI_ISL_15970969	EPI_ISL_15801501	EPI_ISL_15997730	EPI_ISL_15895349
EPI_ISL_15970972	EPI_ISL_15801502	EPI_ISL_15997731	EPI_ISL_15895343
EPI_ISL_15800454	EPI_ISL_15992129	EPI_ISL_15997735	EPI_ISL_15895345
EPI_ISL_15800442	EPI_ISL_15992135	EPI_ISL_15997728	EPI_ISL_15895342
EPI_ISL_15776879	EPI_ISL_15992124	EPI_ISL_15997726	EPI_ISL_15895334
EPI_ISL_15776875	EPI_ISL_15808980	EPI_ISL_15839968	EPI_ISL_15895336
EPI_ISL_15781883	EPI_ISL_15808988	EPI_ISL_15839965	EPI_ISL_15997989
EPI_ISL_15781882	EPI_ISL_15808999	EPI_ISL_15839962	EPI_ISL_15997976
EPI_ISL_15781880	EPI_ISL_15809000	EPI_ISL_15877698	EPI_ISL_15997973
EPI_ISL_15781872	EPI_ISL_15809013	EPI_ISL_15898895	EPI_ISL_15997978
EPI_ISL_15781866	EPI_ISL_15809014	EPI_ISL_15898860	EPI_ISL_15997977
EPI_ISL_15781864	EPI_ISL_15809017	EPI_ISL_15898818	EPI_ISL_15997970
EPI_ISL_15796078	EPI_ISL_15809019	EPI_ISL_15898805	EPI_ISL_15997968
EPI_ISL_15790754	EPI_ISL_15814463	EPI_ISL_15898765	EPI_ISL_15997963

EPI_ISL_15790756	EPI_ISL_15814458	EPI_ISL_15898688	EPI_ISL_15897917
EPI_ISL_15790757	EPI_ISL_15814142	EPI_ISL_15898678	EPI_ISL_15897932
EPI_ISL_15790753	EPI_ISL_15814169	EPI_ISL_15898667	EPI_ISL_15897939
EPI_ISL_15790743	EPI_ISL_15814175	EPI_ISL_15898665	EPI_ISL_15908768
EPI_ISL_15790733	EPI_ISL_15814176	EPI_ISL_15898668	EPI_ISL_15908770
EPI_ISL_15790734	EPI_ISL_15814181	EPI_ISL_15898664	EPI_ISL_15908778
EPI_ISL_15790706	EPI_ISL_15814189	EPI_ISL_15898655	EPI_ISL_15908780
EPI_ISL_15790703	EPI_ISL_15814195	EPI_ISL_15898652	EPI_ISL_15908782
EPI_ISL_15790854	EPI_ISL_15814199	EPI_ISL_15898632	EPI_ISL_15908786
EPI_ISL_15803223	EPI_ISL_15814201	EPI_ISL_15898643	EPI_ISL_15908788
EPI_ISL_15803221	EPI_ISL_15814202	EPI_ISL_15898629	EPI_ISL_15908790
EPI_ISL_15908792	EPI_ISL_15938094	EPI_ISL_15955381	EPI_ISL_15983088
EPI_ISL_15908798	EPI_ISL_15938087	EPI_ISL_15955368	EPI_ISL_15983089
EPI_ISL_15908801	EPI_ISL_15938088	EPI_ISL_15957762	EPI_ISL_15983091
EPI_ISL_15908803	EPI_ISL_15938082	EPI_ISL_15957757	EPI_ISL_15983092
EPI_ISL_15908804	EPI_ISL_15938083	EPI_ISL_15957750	EPI_ISL_15983094
EPI_ISL_15908805	EPI_ISL_15938079	EPI_ISL_15957758	EPI_ISL_15983097
EPI_ISL_15908808	EPI_ISL_15938076	EPI_ISL_15957749	EPI_ISL_15983099
EPI_ISL_15908810	EPI_ISL_15938072	EPI_ISL_15957735	EPI_ISL_15983103
EPI_ISL_15908811	EPI_ISL_15938070	EPI_ISL_15957736	EPI_ISL_15983106
EPI_ISL_15908815	EPI_ISL_15938065	EPI_ISL_15964538	EPI_ISL_15983107
EPI_ISL_15908816	EPI_ISL_15938061	EPI_ISL_15964537	EPI_ISL_15983780
EPI_ISL_15910011	EPI_ISL_15938059	EPI_ISL_15964533	EPI_ISL_15983778
EPI_ISL_15909999	EPI_ISL_15938057	EPI_ISL_15964531	EPI_ISL_15983768
EPI_ISL_15912357	EPI_ISL_15938052	EPI_ISL_15964532	EPI_ISL_15983761
EPI_ISL_15912354	EPI_ISL_15938044	EPI_ISL_15964527	EPI_ISL_15983771
EPI_ISL_15912347	EPI_ISL_15938035	EPI_ISL_15998923	EPI_ISL_15983769
EPI_ISL_15912346	EPI_ISL_15938030	EPI_ISL_15998929	EPI_ISL_15983762
EPI_ISL_15912981	EPI_ISL_15938031	EPI_ISL_15998917	EPI_ISL_15983750
EPI_ISL_15912975	EPI_ISL_15938029	EPI_ISL_15998913	EPI_ISL_15983744
EPI_ISL_15912973	EPI_ISL_15998890	EPI_ISL_15998921	EPI_ISL_15983741
EPI_ISL_15912967	EPI_ISL_15998887	EPI_ISL_15998907	EPI_ISL_15983736
EPI_ISL_15916607	EPI_ISL_15998882	EPI_ISL_15998911	EPI_ISL_15983742
EPI_ISL_15916611	EPI_ISL_15998878	EPI_ISL_15998910	EPI_ISL_15983734
EPI_ISL_15916608	EPI_ISL_15998877	EPI_ISL_15998909	EPI_ISL_15983728
EPI_ISL_15916612	EPI_ISL_15998874	EPI_ISL_15998898	EPI_ISL_15983740
EPI_ISL_15916600	EPI_ISL_15998872	EPI_ISL_15998900	EPI_ISL_15983732
EPI_ISL_15916605	EPI_ISL_15998868	EPI_ISL_15998897	EPI_ISL_16018040
EPI_ISL_15916604	EPI_ISL_15998869	EPI_ISL_15998896	EPI_ISL_15983719
EPI_ISL_15916602	EPI_ISL_15998866	EPI_ISL_15998893	EPI_ISL_15983720
EPI_ISL_15916603	EPI_ISL_15998860	EPI_ISL_15998891	EPI_ISL_15983716
EPI_ISL_15916598	EPI_ISL_15941866	EPI_ISL_15971250	EPI_ISL_15999330
EPI_ISL_15916599	EPI_ISL_15941863	EPI_ISL_15973003	EPI_ISL_15999332
EPI_ISL_15916594	EPI_ISL_15941870	EPI_ISL_15972999	EPI_ISL_15999325
EPI_ISL_15916601	EPI_ISL_15941868	EPI_ISL_15972998	EPI_ISL_15999326
EPI_ISL_15997959	EPI_ISL_15941857	EPI_ISL_15972996	EPI_ISL_15999320

EPI_ISL_15997948	EPI_ISL_15941851	EPI_ISL_15972995	EPI_ISL_15999319
EPI_ISL_15997951	EPI_ISL_15941852	EPI_ISL_15972994	EPI_ISL_15999316
EPI_ISL_15997953	EPI_ISL_15941849	EPI_ISL_15983064	EPI_ISL_15999312
EPI_ISL_15997947	EPI_ISL_15941846	EPI_ISL_15983070	EPI_ISL_15999309
EPI_ISL_15997944	EPI_ISL_16055706	EPI_ISL_15983072	EPI_ISL_15999308
EPI_ISL_15997943	EPI_ISL_16055702	EPI_ISL_15983073	EPI_ISL_16003262
EPI_ISL_15997937	EPI_ISL_15950020	EPI_ISL_15983074	EPI_ISL_16003260
EPI_ISL_15997936	EPI_ISL_15950008	EPI_ISL_15983075	EPI_ISL_16004464
EPI_ISL_15938091	EPI_ISL_15950004	EPI_ISL_15983082	EPI_ISL_16004459
EPI_ISL_15938090	EPI_ISL_15949996	EPI_ISL_15983085	EPI_ISL_16008333
EPI_ISL_15938097	EPI_ISL_15949995	EPI_ISL_15983087	EPI_ISL_16008336
EPI_ISL_16008332	EPI_ISL_16017647	EPI_ISL_16055377	EPI_ISL_16080102
EPI_ISL_16008335	EPI_ISL_16020312	EPI_ISL_16055378	EPI_ISL_16082199
EPI_ISL_16008325	EPI_ISL_16020313	EPI_ISL_16055374	EPI_ISL_16093925
EPI_ISL_16008328	EPI_ISL_16020316	EPI_ISL_16055698	EPI_ISL_16093924
EPI_ISL_16008323	EPI_ISL_16020307	EPI_ISL_16055691	EPI_ISL_16093922
EPI_ISL_16008318	EPI_ISL_16020303	EPI_ISL_16055690	EPI_ISL_16093914
EPI_ISL_16008320	EPI_ISL_16020294	EPI_ISL_16093760	EPI_ISL_16093919
EPI_ISL_16008313	EPI_ISL_16020292	EPI_ISL_16093751	EPI_ISL_16093915
EPI_ISL_16017262	EPI_ISL_16020288	EPI_ISL_16093752	EPI_ISL_16093912
EPI_ISL_16017265	EPI_ISL_16020287	EPI_ISL_16093746	EPI_ISL_16093911
EPI_ISL_16017258	EPI_ISL_16020284	EPI_ISL_16093742	EPI_ISL_16093909
EPI_ISL_16017251	EPI_ISL_16020283	EPI_ISL_16093738	EPI_ISL_16093935
EPI_ISL_16017247	EPI_ISL_16020281	EPI_ISL_16093735	EPI_ISL_16122503
EPI_ISL_16017245	EPI_ISL_16020282	EPI_ISL_16093733	EPI_ISL_16122497
EPI_ISL_16017231	EPI_ISL_16020628	EPI_ISL_16093732	EPI_ISL_16122494
EPI_ISL_16017225	EPI_ISL_16020630	EPI_ISL_16093729	EPI_ISL_16122489
EPI_ISL_16017227	EPI_ISL_16020635	EPI_ISL_16093727	EPI_ISL_16122485
EPI_ISL_16017226	EPI_ISL_16020636	EPI_ISL_16093724	EPI_ISL_16122475
EPI_ISL_16017224	EPI_ISL_16020640	EPI_ISL_16093717	EPI_ISL_16122458
EPI_ISL_16017221	EPI_ISL_16020642	EPI_ISL_16093716	EPI_ISL_16122452
EPI_ISL_16017219	EPI_ISL_16020650	EPI_ISL_16093709	EPI_ISL_16122449
EPI_ISL_16017211	EPI_ISL_16036633	EPI_ISL_16093701	EPI_ISL_16122446
EPI_ISL_16017208	EPI_ISL_16052032	EPI_ISL_16093699	EPI_ISL_16122441
EPI_ISL_16017202	EPI_ISL_16052033	EPI_ISL_16093700	EPI_ISL_16122444
EPI_ISL_16017203	EPI_ISL_16052035	EPI_ISL_16093698	EPI_ISL_16122431
EPI_ISL_16017200	EPI_ISL_16052037	EPI_ISL_16093695	EPI_ISL_16122428
EPI_ISL_16018031	EPI_ISL_16052038	EPI_ISL_16093696	EPI_ISL_16122419
EPI_ISL_16018575	EPI_ISL_16052039	EPI_ISL_16093692	EPI_ISL_16122410
EPI_ISL_16018577	EPI_ISL_16052047	EPI_ISL_16093694	EPI_ISL_16122400
EPI_ISL_16018568	EPI_ISL_16052060	EPI_ISL_16077171	EPI_ISL_16122389
EPI_ISL_16018567	EPI_ISL_16052061	EPI_ISL_16077167	EPI_ISL_16122366
EPI_ISL_16018564	EPI_ISL_16052065	EPI_ISL_16077169	EPI_ISL_16122365
EPI_ISL_16018559	EPI_ISL_16055404	EPI_ISL_16077162	EPI_ISL_16122363
EPI_ISL_16018558	EPI_ISL_16055411	EPI_ISL_16077166	EPI_ISL_16122359
EPI_ISL_16018560	EPI_ISL_16055401	EPI_ISL_16077159	EPI_ISL_16122356

EPI_ISL_16018553	EPI_ISL_16055402	EPI_ISL_16077157	EPI_ISL_16122346
EPI_ISL_16018550	EPI_ISL_16055397	EPI_ISL_16077155	EPI_ISL_16122329
EPI_ISL_16018554	EPI_ISL_16055393	EPI_ISL_16077153	EPI_ISL_16122326
EPI_ISL_16018555	EPI_ISL_16055394	EPI_ISL_16077149	EPI_ISL_16122331
EPI_ISL_16018551	EPI_ISL_16055392	EPI_ISL_16077148	EPI_ISL_16122332
EPI_ISL_16018548	EPI_ISL_16055391	EPI_ISL_16077144	EPI_ISL_16122328
EPI_ISL_16018549	EPI_ISL_16055389	EPI_ISL_16077142	EPI_ISL_16122325
EPI_ISL_16018547	EPI_ISL_16055388	EPI_ISL_16077140	EPI_ISL_16122319
EPI_ISL_16017515	EPI_ISL_16055386	EPI_ISL_16077136	EPI_ISL_16122320
EPI_ISL_16017512	EPI_ISL_16055381	EPI_ISL_16080104	EPI_ISL_16122296
EPI_ISL_16017500	EPI_ISL_16055379	EPI_ISL_16080099	EPI_ISL_16122293
EPI_ISL_16122282			
EPI_ISL_16122258			
EPI_ISL_16122236			
EPI_ISL_16122233			
EPI_ISL_16122238			
EPI_ISL_16122208			
EPI_ISL_16122200			
EPI_ISL_16122161			
EPI_ISL_16122156			
