# Supplementary material for: Selective Pressure and Evolution of SARS-CoV-2 Lineages BF.7 and BQ.1.1 Circulating in Italy from July to December 2022
Source: Microorganisms. 2024 Apr 30;12(5):908. doi: 10.3390/microorganisms12050908 (PMC11124320; doi:10.3390/microorganisms12050908)
Supplement: Supplementary file 1 [file microorganisms-12-00908-s001.zip › Table S2.pdf]

Table S2. Selective pressure analysis on SARS-CoV-2 lineages BF.7 and BQ.1.1

	nsp1 (BF.7)	***nsp1 (BQ.1.1)***
Positively selected sites (w for sites > 1) HYPHY	/	/
Negatively selected sites (w for sites < 1) HYPHY	/	85 (M,V)
	nsp2 (BF.7)	***nsp2 (BQ.1.1)***
Positively selected sites (w for sites > 1) HYPHY	/	/
Negatively selected sites (w for sites < 1) HYPHY	630 (N)	/
	nsp3 (BF.7)	***nsp3 (BQ.1.1)***
Positively selected sites (w for sites > 1) HYPHY	180 (Q, H); 1329 (N; D)	/
Negatively selected sites (w for sites < 1) HYPHY	135 (D); 1445 (C); 1591 (C); 1594 (C); 1603 (V)	79 (L); 460 (F); 1741 (L; F)
	nsp4 (BF.7)	***nsp4 (BQ.1.1)***
Positively selected sites (w for sites > 1) HYPHY	/	/
Negatively selected sites (w for sites < 1) HYPHY	290 (V)	223 (H); 290 (V)
	nsp5 (BF.7)	***nsp5 (BQ.1.1)***
Positively selected sites (w for sites > 1) HYPHY	/	/
Negatively selected sites (w for sites < 1) HYPHY	48 (D); 216 (D)	/
	nsp6 (BF.7)	***nsp6 (BQ.1.1)***
Positively selected sites (w for sites > 1) HYPHY	/	/

Negatively selected sites (w for sites < 1) HYPHY	133 (D)	/
	nsp7 (BF.7)	***nsp7 (BQ.1.1)***
Positively selected sites (w for sites > 1) HYPHY	/	/
Negatively selected sites (w for sites < 1) HYPHY	/	/
	nsp8 (BF.7)	***nsp8 (BQ.1.1)***
Positively selected sites (w for sites > 1) HYPHY	/	/
Negatively selected sites (w for sites < 1) HYPHY	/	/
	nsp9 (BF.7)	***nsp9 (BQ.1.1)***
Positively selected sites (w for sites > 1) HYPHY	/	/
Negatively selected sites (w for sites < 1) HYPHY	/	105 (S)
	nsp10 (BF.7)	***nsp10 (BQ.1.1)***
Positively selected sites (w for sites > 1) HYPHY	/	/
Negatively selected sites (w for sites < 1) HYPHY	113 (K)	/
	nsp11 (BF.7)	***nsp11 (BQ.1.1)***
Positively selected sites (w for sites > 1) HYPHY	/	/
Negatively selected sites (w for sites < 1) HYPHY	/	/
	nsp12 (BF.7)	***nsp12 (BQ.1.1)***
Positively selected sites	/	/

(w for sites > 1) HYPHY		
Negatively selected sites (w for sites < 1) HYPHY	105 (R)	/
	nsp13 (BF.7)	***nsp13 (BQ.1.1)***
Positively selected sites (w for sites > 1) HYPHY	/	/
Negatively selected sites (w for sites < 1) HYPHY	128 (E); 202 (K); 390 (R); 462 (K); 560 (R)	/
	nsp14 (BF.7)	***nsp14 (BQ.1.1)***
Positively selected sites (w for sites > 1) HYPHY	/	/
Negatively selected sites (w for sites < 1) HYPHY	41 (D); 53 (R); 485 (R)	/
	nsp15 (BF.7)	***nsp15 (BQ.1.1)***
Positively selected sites (w for sites > 1) HYPHY	/	/
Negatively selected sites (w for sites < 1) HYPHY	/	79 (I); 218 (M, T); 230 (Y)
	nsp16 (BF.7)	***nsp16 (BQ.1.1)***
Positively selected sites (w for sites > 1) HYPHY	/	/
Negatively selected sites (w for sites < 1) HYPHY	2 (S); 7 (P)	7 (P); 199 (A)
	ORF3a (BF.7)	***ORF3a (BQ.1.1)***
Positively selected sites (w for sites > 1) HYPHY	/	/
Negatively selected sites (w for sites < 1) HYPHY	/	130 (C)
	E (BF.7)	***E (BQ.1.1)***

Positively selected sites (w for sites > 1) HYPHY	/	/
Negatively selected sites (w for sites < 1) HYPHY	/	/
	M (BF.7)	***M (BQ.1.1)***
Positively selected sites (w for sites > 1) HYPHY	/	/
Negatively selected sites (w for sites < 1) HYPHY	/	/
	N (BF.7)	***N (BQ.1.1)***
Positively selected sites (w for sites > 1) HYPHY	/	/
Negatively selected sites (w for sites < 1) HYPHY	158 (V); 309 (P)	/
	ORF6 (BF.7)	***ORF6 (BQ.1.1)***
Positively selected sites (w for sites > 1) HYPHY	/	/
Negatively selected sites (w for sites < 1) HYPHY	/	/
	ORF7a (BF.7)	***ORF7a (BQ.1.1)***
Positively selected sites (w for sites > 1) HYPHY	/	/
Negatively selected sites (w for sites < 1) HYPHY	79 (A)	/
	ORF7b (BF.7)	***ORF7b (BQ.1.1)***
Positively selected sites (w for sites > 1) HYPHY	/	/
Negatively selected sites (w for sites < 1) HYPHY	/	/

	ORF8 (BF.7)	***ORF8 (BQ.1.1)***
Positively selected sites (w for sites > 1) HYPHY	/	121 (I; L; T)
Negatively selected sites (w for sites < 1) HYPHY	/	/
	ORF10 (BF.7)	***ORF10 (BQ.1.1)***
Positively selected sites (w for sites > 1) HYPHY	/	/
Negatively selected sites (w for sites < 1) HYPHY	/	/
	Spike (BF.7)	***Spike (BQ.1.1)***
Positively selected sites (w for sites > 1) HYPHY	408 (R, S); 440 (N; K)	452 (L; R)
Negatively selected sites (w for sites < 1) HYPHY	138 (D); 146 (H, Y); 508 (Y); 619 (E)	457 (R); 1216 (I)
